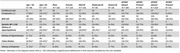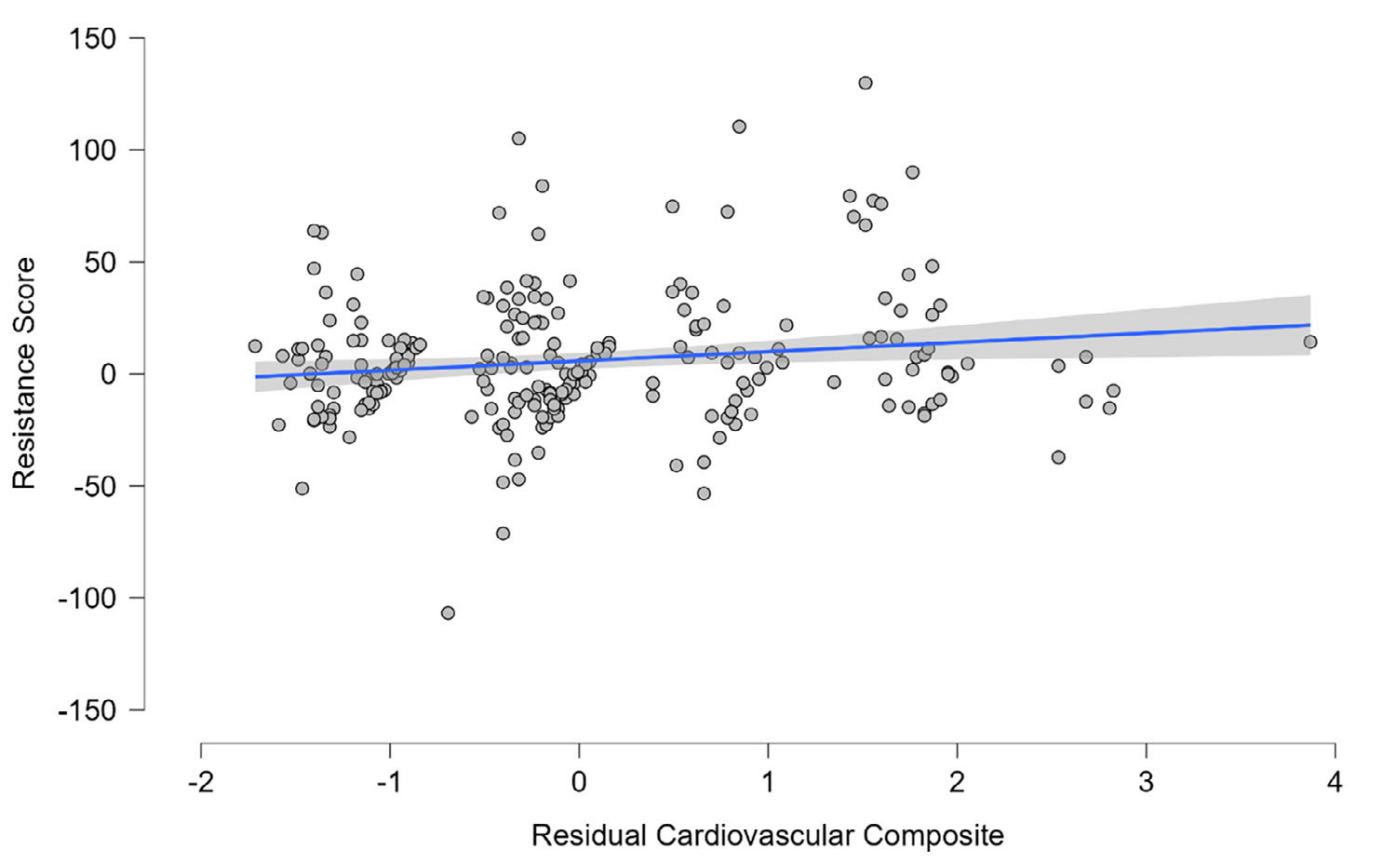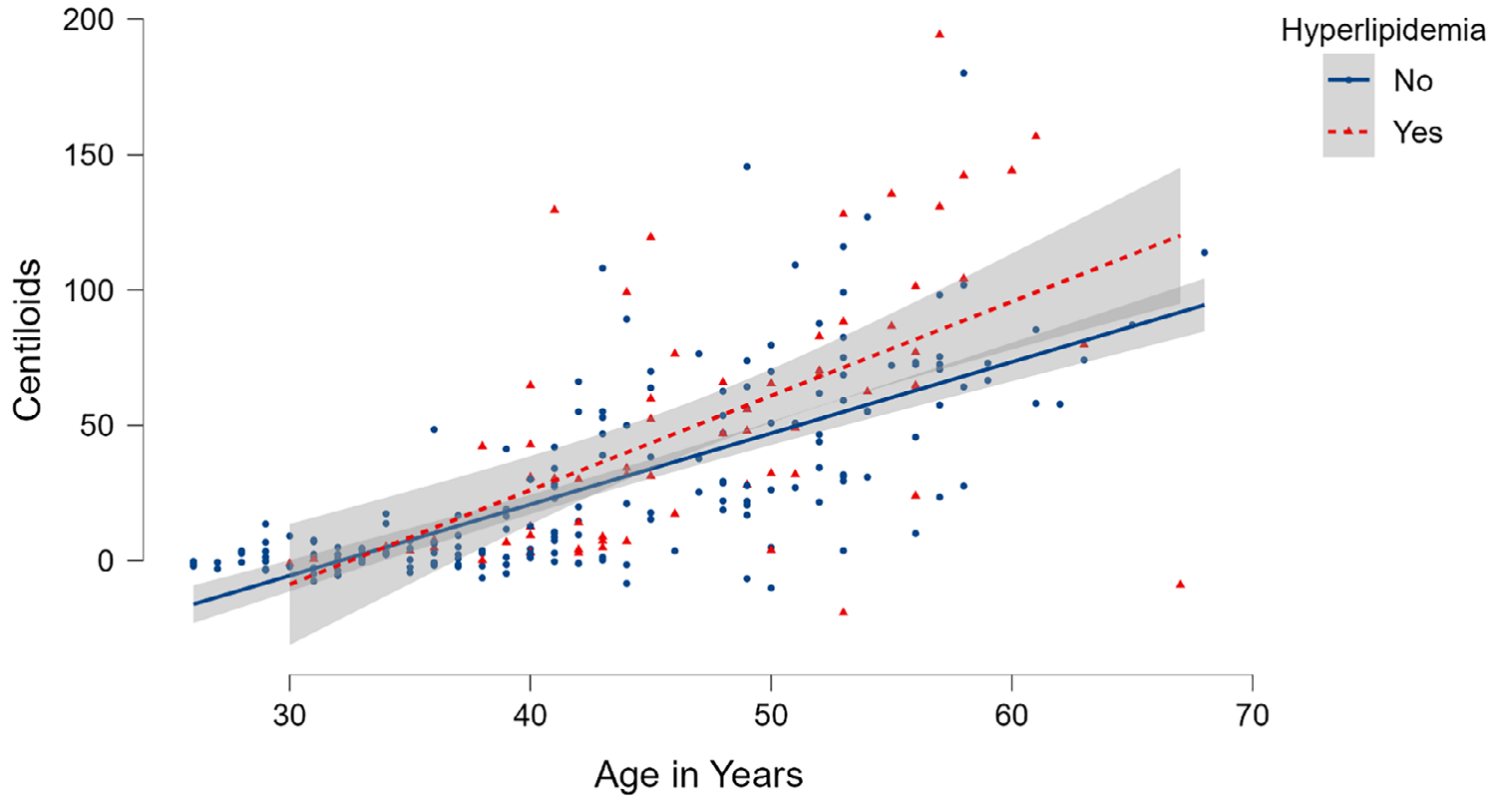# Cardiovascular Risk Composite and Time of Amyloid Accumulation and Cognitive Decline in Down Syndrome Alzheimer's Disease

**DOI:** 10.1002/alz70857_099395

**Published:** 2025-12-24

**Authors:** Courtney Brothers, Julie K. Wisch, Benjamin L Handen, Patrick J. Lao, Beau Ances, James Tyler Kennedy, Matthew D Zammit, Bradley T Christian, Wayne Silverman, Elizabeth Head, Joseph H. Lee, Adam Brickman, Charles K Stone

**Affiliations:** ^1^ University of Wisconsin‐Madison, Madison, WI, USA; ^2^ Washington University in St. Louis School of Medicine, St. Louis, MO, USA; ^3^ University of Pittsburgh, Pittsburgh, PA, USA; ^4^ Columbia University Irving Medical Center, New York, NY, USA; ^5^ Taub Institute for Research on Alzheimer's Disease and the Aging Brain, Vagelos College of Physicians and Surgeons, Columbia University, New York, NY, USA; ^6^ Washington University School of Medicine, St. Louis, MO, USA; ^7^ Knight Alzheimer Disease Research Center, St. Louis, MO, USA; ^8^ Waisman Center, University of Wisconsin‐Madison, Madison, WI, USA; ^9^ Department of Medical Physics, University of Wisconsin‐Madison, Madison, WI, USA; ^10^ Department of Medical Physics, University of Wisconsin, Madison, WI, USA; ^11^ Wisconsin Alzheimer's Disease Research Center, University of Wisconsin‐Madison School of Medicine and Public Health, Madison, WI, USA; ^12^ University of California, Irvine, Irvine, CA, USA; ^13^ Taub Institute for Research on Alzheimer's Disease and the Aging Brain, Columbia University, New York, NY, USA; ^14^ University of Wisconsin‐Madison School of Medicine and Public Health, Madison, WI, USA

## Abstract

**Background:**

Individuals with Down syndrome (DS) have a 90% lifetime risk for symptomatic Alzheimer's disease. Yet, there is a 20+ year age range in when individuals with DS reach PET amyloid‐beta (Aβ) positivity (i.e., centiloid (CL) ≥18). There is a critical need to identify factors associated with resistance (i.e., less than expected Aβ for age) and resilience (i.e., better than expected cognitive function given Aβ) in the DS community.

**Method:**

262 adults with DS (M=43.93, SD=9.29) in the Alzheimer Biomarker Consortium‐Down syndrome underwent MRI and amyloid PET scans with [11C] PiB or [18F] florbetapir. A study partner reported on health history. Systolic blood pressure and BMI were obtained.

Using multiple regression models, we examined the best fit function for association between: 1) age and Aβ, and 2) Aβ and cognitive performance, adjusted for premorbid intellectual disability level. We than calculated each participant's residual (i.e., difference between observed versus predicted value) to assess *resistance to Aβ* (defined as less than predicted Aβ for age) and *resilience to cognitive impairment* (defined as better than predicted cognition for Aβ). The modified Cued Recall Test and Down Syndrome Mental Status Exam were used as cognitive scores. The cardiovascular composite was based on the original and no‐lab Framingham Risk Score (BMI ≥ 30, systolic blood pressure ≥ 130, use of statins, and presence of hypertension, hyperlipidemia, and diabetes).

**Result:**

In partial correlations adjusted for age, there was a significant positive association between the cardiovascular composite (*r* = .156, p = .016) and *resistance* score; higher cardiovascular composite was associated with greater than expected Aβ for age. At the item level, hyperlipidemia was associated with a 1.8 x higher likelihood of greater than expected Aβ for age (OR = 1.83, p = .047). There was not a significant correlation between the cardiovascular composite and the *resilience* scores.

**Conclusion:**

Cardiovascular conditions may alter resistance to DSAD, including an earlier age of Aβ accumulation, and be an important target for delaying or preventing DSAD.